# Subjective Feelings of Polish Doctors after Receiving the COVID-19 Vaccine

**DOI:** 10.3390/ijerph18126291

**Published:** 2021-06-10

**Authors:** Krzysztof Zdziarski, Marek Landowski, Paulina Zabielska, Beata Karakiewicz

**Affiliations:** 1 Subdepartment of Social Medicine and Public Health, Department of Social Medicine, Pomeranian Medical University in Szczecin, 71-210 Szczecin, Poland; krzysztof.zdziarski@pum.edu.pl (K.Z.); beata.karakiewicz@pum.edu.pl (B.K.); 2 Department of Computer Science, Faculty of Computer Science and Telecommunications, Maritime University of Szczecin, 70-500 Szczecin, Poland; m.landowski@am.szczecin.pl

**Keywords:** COVID-19, vaccine, doctor, subjective feelings, psychosocial condition, attitude, behaviour

## Abstract

The COVID-19 pandemic has caused enormous confusion around the world in our daily existence. The security measures taken, such as physical distance, wearing a mask, quarantine or closing shopping malls, and even isolating large groups of the population, did not contribute to the complete overcoming of the problem. Information on the positive results of research into the COVID-19 vaccine and, finally, its administration offered hope that the pandemic would be overcome. The undertaken problem of research concerning the subjective analysis of the feelings of doctors in Poland after receiving the COVID-19 vaccine shows an important area of the medical world, which is directly responsible for their own health and the patients entrusted to them. In addition, 149 people participated in the study (8–13 February 2021), including 57% of women and 43% of men. The minimum and maximum age of the respondents are 26 and 69 years old. Furthermore, 85% of respondents took two doses of the vaccine and 15% took one. The authorial questionnaire was completed by the participants in the study online in February 2021. The results obtained indicate that the COVID-19 vaccine generates hope for stopping the pandemic. In addition, 96% of research participants think so. Doctors in middle and mature age are the most optimistic, while the youngest ones are less optimistic. In addition, 57% of respondents do not worry about side effects after taking the vaccine. Fear at the time of vaccination was experienced by over 9% of doctors. The most frequently reported post-vaccination reactions are injection site pain, fatigue and headache. Increased temperature occurred in older female respondents. From a psychosocial perspective, men are more likely to fear being infected personally with the virus, and women are more likely to be infected with their loved ones. The presented subjective assessment presents the physicians’ view captured at the moment in terms of existential and emotional. The presented feelings of the research participants reflect their personal satisfaction, responsibility for their own health, care for their relatives and patients.

## 1. Introduction

On the one hand, research on vaccine efficacy conducted by scientists is optimistic and, on the other hand, suggests doubts, which in turn may delay the vaccination process. The results of laboratory studies on the effectiveness of vaccines indicate 90% vaccine efficacy. Skeptics of this view emphasize that this result is achievable when the preparations are tested under ideal clinical conditions and when the transmission of infection is lower. With a higher number of infections, the effectiveness of the preparations is not satisfactory [1]. Other studies show that new vaccines should not be waited more efficiently as this may cause the pandemic to spread more widely [2]. Doctors in Poland and many other countries belong to the so-called the zero group that is the first to receive the vaccine. Taking vaccines by doctors is an important factor that stimulates the society to follow attitudes and at the same time strengthens respondents in their existential and psychological condition. From a social point of view, this is extremely important because, in general, people tend to express negative emotions, resentment or fear during a pandemic [3,4]. Doctors have been exposed to anxiety and depression from the very beginning of the pandemic [5]. This is confirmed by the results of studies conducted on this issue, which indicate a high state of anxiety and depression during a pandemic [6]. Other studies confirm that doctors treating patients with COVID-19 are at high risk for mental health problems. In China, about 14% of physicians showed moderate to severe depressive symptoms, and, in Wuhan alone, severe symptoms of depression, anxiety, fear and insomnia [7]. The above behaviors are also associated with occupational burnout, which, according to reports from many studies, is at a high level [8]. Taking the vaccine may have a positive effect on the psychosocial behavior of respondents, which translates not only into their personal well-being, but also into positive interactions with patients. The subjective opinions presented provide views on the effectiveness of the vaccine, personal satisfaction, and the effectiveness of vaccination to deal with the pandemic. They also present existential attitudes in the face of the prevailing pandemic, which are related to caring for loved ones and oneself. Moreover, they reveal the level of personal fear and of their families fear of becoming infected with COVID-19.

The feelings of the respondents after receiving vaccinations express their personal inner opinion from the perspective of their subjective sense of psychological comfort and well-being. The subjective approach used in the research is applicable to cognitive-behavioral concepts that emphasize the role of cognitive processes, which make it possible to register and become aware of what a person feels, thinks and experiences at a given moment, not neglecting the importance of emotions, which constitute the content of each cognition [9]. The undertaken research problem focuses on presenting the subjective feelings of Polish doctors after receiving the COVID-19 vaccine. The information obtained is important for the psychosocial functioning of physicians because the pandemic increases the demands placed on them and puts them at high risk of adversely affecting mental health [10].

## 2. Materials and Methods

The study was conducted using a proprietary questionnaire, including 17 questions that were asked to doctors in electronic form. The first part of the questionnaire included sociodemographic questions that were asked to doctors in electronic form. The next questions were:

1. How many doses of the vaccine have you taken?
2. Did you take the vaccine voluntarily?
3. Did you feel fear when you were vaccinated?
4. Are you concerned about the negative effects of the vaccine?
5. Did you need a doctor’s help after receiving the vaccine?
6. Have you been hospitalized after receiving the vaccine?
7. Have there been any post-vaccination reactions? (injection site pain, swelling, redness, fatigue, headache, muscle pain, joint pain, chills, fever, nausea).
8. How do you rate the effectiveness of the vaccine you have received?
9. Will taking the vaccine help to contain the pandemic?
10. Are you afraid of getting infected with COVID-19?
11. Are you afraid of infection and illness of someone close to you?
12. Did any of your family have COVID-19?
13. Did any of your family die from COVID-19?

The research tool was created for the needs of the current pandemic situation to collect data for further exploration on an ongoing basis. The studied sample consisted of 149 people selected on purpose during the course that was to improve the qualification. The respondents completed an electronic questionnaire using Microsoft Teams. The response rate was 100%. Quantitative data are given as mean ± standard deviation and percentages. The Mann–Whitney test, Kruskal–Wallis test and chi-square test for independence were used to analyze the differences between the distributions. The Pearson correlation coefficient was used to test the correlation. A significance level of 0.05 was adopted for all tests.

## 3. Results

The respondents were active doctors, women and men, after receiving the COVID-19 vaccine. Of those surveyed, 85% (127 people) received two doses of the vaccine, the remaining 15% (22 people) received one dose of the vaccine. Women constituted 57% and men 43%. The mean and standard deviation of the age of the respondents were 32.1 ± 6.99, and the median, mode, minimum and maximum age were respectively: 29, 28, 26 and 69 years. Moreover, 62.4% of the respondents (93 people) were in the 26–30 age group, 32.2% in the 31–45 age group and 5.4% in the 46–69 age group. The mean standard deviation of the respondents’ work experience was 6.2 ± 6.8. The correlation between the length of service and the age of the respondents is positively very high and the Pearson correlation coefficient is 0.97 (*p* < 0.001). The mean and standard deviation of age and length of service, broken down into individual age groups and gender, are presented in Table 1.

The results obtained from the conducted research indicate that 96% of the respondents (143 people) agree with the opinion that taking the vaccine will contribute to stopping the pandemic. Using the chi-square test for independence, the ratio of respondents to the vaccine in terms of age and gender was examined, and it was determined whether the obtained results were dependent on the respondents’ age and gender. In two cases, it was noticed that the answers depended on the age of the respondents, i.e., in the question about the assessment of vaccine effectiveness (*p* = 0.012) and the fear of negative effects after vaccination (*p* = 0.027). In the group of the youngest respondents (26–30 years of age), over 50% of respondents from this group indicated no opinion on the effectiveness of the vaccine, and 47.3% of people assessed the effectiveness of the vaccine as positive.

In the older age groups (31–45 and 46–69), the vast majority (over 75% of people in a given group) assess the effectiveness of the vaccine as satisfactory. This research has shown that 35% of respondents in the 31–45 age group are concerned about the negative effects of the vaccine. In the 26–30 and 46–69 age groups, this opinion is expressed by 12.5% of physicians.

In the remaining cases, as regards the age of the respondents and their gender, the chi-square test did not show any significant differences in the answers to individual questions.

Table 2 and Table 3 present a comparison of the respondents’ answers depending on age and gender.

The collected research results confirm that 58.4% (87 people) of the respondents consider the vaccine to be effective. In addition, 39.6% of respondents (59 people) did not have an opinion on this subject; 2% of respondents (3 people) consider the vaccine to be ineffective; 57% of respondents (85 people) are not afraid of side effects after taking the vaccine; 18.8% (28 people) feel such fear; and 20.1% of respondents (30 people) do not think about it. This fact is indifferent to 4% of respondents (6 people). Fear during vaccination was experienced by 9.4% of respondents (14 people), while 32.9% (49 people) of respondents were afraid of being vaccinated. Furthermore, 57.7% (86 people) did not feel fear at all during vaccination.

**Figure 1:**
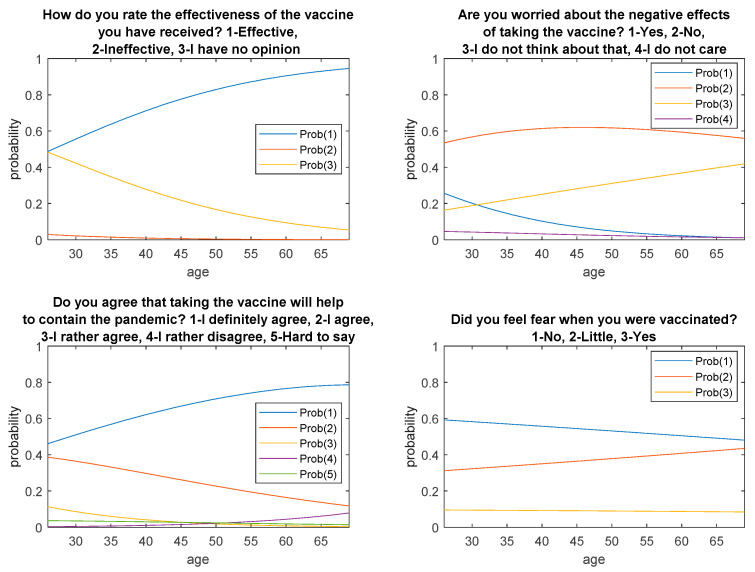
Probability distributions obtained from logistic regression concerning respondents’ answers depending on age.

**Figure 2:**
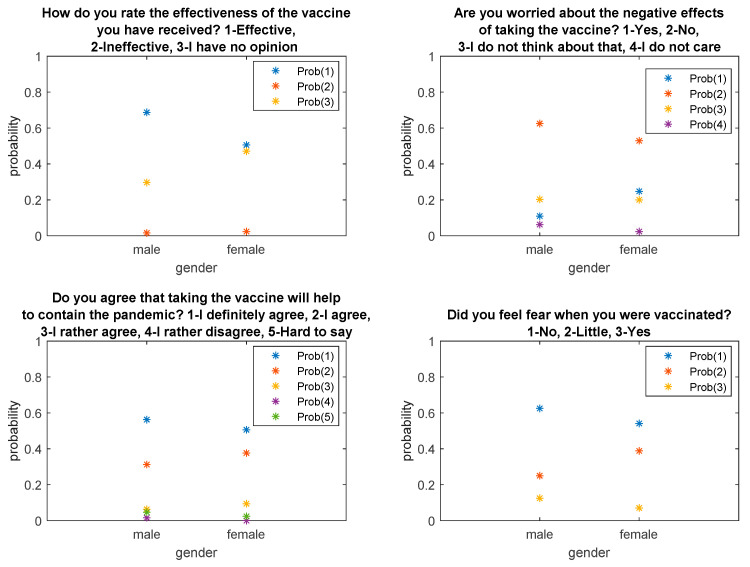
Probability distributions concerning respondents’ answers depending on gender.

Figure 1 and Figure 2 show fitted probabilities obtained with multinomial logistic regression. The presented distributions confirm the above-described relationships regarding the respondents’ answers depending on age and gender.

The results of the study also showed what the side effects are after the administration of the vaccine. Only one person (0.7%) confirmed that they did not experience any side effects. Other respondents most often reported the following symptoms as side effects: pain at the injection site 94% (140 respondents), fatigue 47.7% (71 people), headache 44.3% (66 people), muscle pain 42.3% (63 people), chills 28.2% (42 people), fever 25.5% (38 people), joint pain 20.8% (31 people), redness or swelling at the injection site 18.8% (28 people) and nausea 8.7% (13 people). None of the respondents needed a doctor’s help after receiving the vaccine. The chi-square test of independence was performed to investigate the relationship between vaccine side effects and age and gender. The tests showed that there is a correlation between the age of the respondents and the temperature after taking the vaccine (*p* = 0.004). Increased temperature was more common in older people aged 46–69 years old in 75% of cases, in the age of 31–45 in 25% of cases, and in the age of 26–30 in 21.5% of cases (Table 4).

The chi-square test also showed a relationship between pain at the injection site and gender of the respondents (*p* = 0.029). Women indicated this side effect more often (97.6%) than men (89.1%). Table 4 presents side effects after administration of the vaccine, which did not show significant differences in individual age groups or by gender.

**Figure 3:**
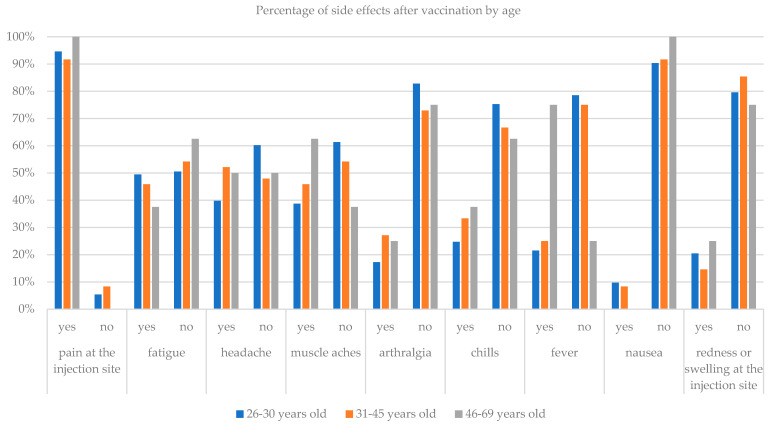
Comparison of the number of side effects after taking the vaccine with age.

**Figure 4:**
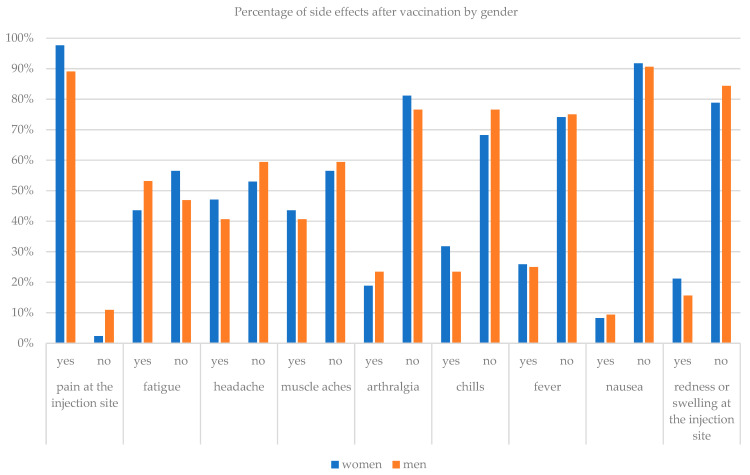
Comparison of the number of side effects after taking the vaccine with gender.

Figure 3 and Figure 4 show histograms of the percentage occurrence of a side effect after administration of the vaccine depending on the age and gender of the respondents.

The conducted research also showed the psychosocial feelings of doctors in the area of fear of infection with the virus and disease towards themselves and their relatives. Answers to the questions were given using the Likert scale from 1 to 5, where 1 means no fear, while 5 is a strong fear of infection and getting sick. Cronbach’s alpha value for these questions is 71%, so the overall reliability of the questionnaire has been met. The correlation for these two questions is moderately positive, and Pearson’s correlation coefficient is 0.59 (*p* < 0.001). The arithmetic mean and standard deviation for questions about fear for oneself and about one’s relatives against virus infection and disease were 3.07 ± 1.32, 4.28 ± 0.89, respectively. To compare the answers of women and men, the Mann–Whitney test was carried out, which showed differences between the respondents’ answers to the question about the fear of self-infection and the COVID-19 disease. The mean response value and standard deviation were 2.69 ± 1.11 and 3.58 ±1.41, respectively. Women fear infection and disease less than men. The level of fear of infection and the disease of a loved one is similar among women and men. The results are presented in Table 5.

A Kruskal–Wallis test and Mann–Whitney test were performed by analyzing the respondents’ answers to the questions concerning fear of infection and disease in terms of age. For the Kruskal–Wallis test, in both cases (fear for oneself and for one’s relatives), the *p*-value was <0.05, which suggests that one or more answers for each age group differ significantly. The Mann–Whitney test showed that differences in responses to the question about fear of infection and disease occurred between the age group of 26–30 years and 31–45 years (*p* < 0.001) and between the age groups of 26–30 years and 46–69 years (*p* = 0.009). People aged 26–30 are less afraid of infection and disease than older people. Regarding the question of fear of infection and the disease of a loved one, differences in responses occurred between the age groups of 26–30 years and 31–45 years (*p* = 0.0013). Respondents aged 31–45 feel a greater fear of getting sick and ill than those aged 26–30. No significant differences were found between the responses for the remaining age groups. The test results as well as the mean values and standard deviation are presented in Table 6.

**Figure 5:**
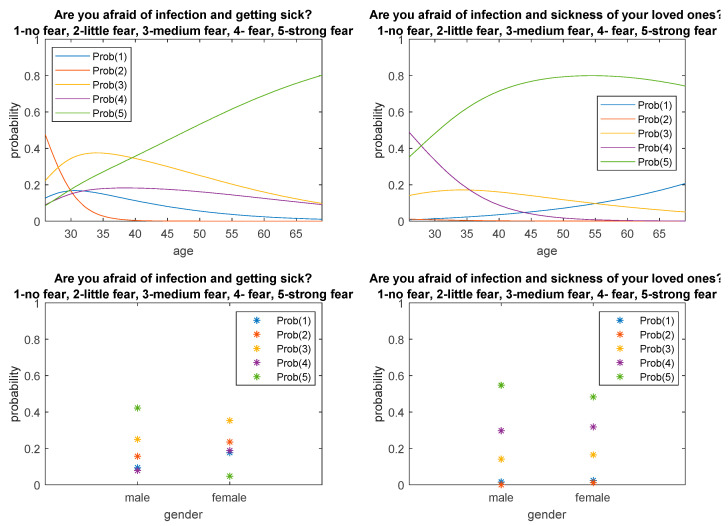
Response probability distributions depending on the age and sex of the respondents.

Figure 5 shows the distribution of probability of respondents’ responses depending on age and gender. The presented probability distributions confirm the above conclusions.

## 4. Discussion

The aim of this study was to show the subjective feelings of Polish doctors after receiving the COVID-19 vaccine. The obtained results confirm the optimistic attitude of the respondents towards their own vaccinations, who are convinced that the pandemic can be stopped in this way. The view was observed in 96% of the study participants. This opinion corresponds with the results research from Italy, which indicate that the COVID-19 vaccine may be the only way to end the pandemic [11]. Our own research conducted shows that opinions on the effectiveness of vaccines are divided and depend on the age of doctors. Middle-aged and mature people (75%) believe that the vaccinations will bring satisfactory results. Young doctors, who constitute a minority in the studied population, are not entirely convinced about the effectiveness of the applied vaccines. However, research has shown some dissonance. Middle-aged doctors (31–45) who had previously stated that vaccinations would work as expected replied that they were concerned about the negative effects of the vaccine (35% of this age group). This position corresponds with other studies that show that the vaccine may be 80% effective when 75% of a given population is vaccinated [12]. Studies have shown that more than half of the surveyed population considered the vaccine to be effective. From an existential point of view, the above opinion raises hope for an improvement in the psychosocial situation of doctors, who, according to other studies, experienced a deterioration in mental well-being due to isolation, anxiety, emotional exhaustion and poor social support during the pandemic [13]. The above thesis is confirmed by research conducted among doctors in Germany [14], where their existential disadvantage was observed during the pandemic. More than 60% of respondents there had concerns about their own health, which were more severe in women. It is noteworthy that, in a review of the literature on the efficacy of the COVID-19 vaccine with 30 articles, eight included healthcare professionals (doctors, nurses, others). The lowest vaccine acceptance was in the Democratic Republic of Congo: 27.7% and the highest in Israel: 78.1% [15]. Interesting research on physicians’ attitudes towards vaccination with the COVID-19 vaccine was also carried out in China. The results showed that 80% of physicians are pro-vaccine if the vaccine is free [16]. The above research reports are optimistic about the death rate of doctors, especially at the beginning of 2020. At that time, there was a huge increase in the mortality of doctors, especially in India [17].

Our own research conducted also included questions related to the feeling of fear during vaccination. The results show that few participants experienced a sense of fear, and almost 58% of the respondents did not feel it at all. The above behaviors may indicate not only a natural approach to the injection procedure, but also a human need to strengthen the condition of doctors to continue functioning in their daily work for the benefit of patients and personal safety. The benefits of vaccine adoption could help mitigate the risks that frontline doctors infected with COVID-19 in the UK and the US have exposed. The lack of appropriate personal protective equipment and an appropriate strategy to protect health care workers from the virus are the most frequent allegations of respondents to local studies [18]. The conducted research showed that more than half of the doctors (57%) are not afraid of side effects after taking the vaccine, while almost every fourth respondent experienced such a condition. On the basis of the data obtained, undesirable behavior after administration of the vaccine can also be indicated. The most frequently revealed complaint was pain at the injection site, which was reported by over 90% of respondents, more often women. Fatigue, headache and muscle pain are other ailments indicated by almost every second respondent. Almost every fourth had chills, joint pain and increased temperature, which was experienced most often by the oldest respondents, and less often by the youngest. Redness at the injection site occurred in almost one in five participants. None of the recipients of the vaccine needed the help of a doctor. The obtained results can be compared with experimental studies in a controlled group, in which the first part of the patients received the vaccine and the second the placebo. Interestingly, undesirable behaviors such as injection site pain, fatigue and headache occurred in 10 placebo recipients and one recipient of the correct vaccine. Overall, the incidence of adverse events was low and similar in both study groups [1]. In contrast, other experimental studies in the US found that, in people who underwent randomization, systemic events were dose-dependent, larger after the second, and smaller after the first. Symptoms were most severe on day 2 and resolved by day 7 after vaccination [19]. The collected material from our own research also revealed feelings about the fear of being personally infected with COVID-19 and the fear of being infected with the virus by loved ones. It has been noted that men are more likely to fear infection. With regard to their relatives, both men and women exhibit similar behaviors before infection and disease. Taking into account the age of the survey participants, it was noticed that older respondents are more afraid of such situations, and younger respondents approach this problem from a distance. Fear of infection and disease for doctors can have negative consequences that translate into interactions with patients and loved ones. Researchers studying various behaviors occurring during a pandemic talk about the emergence of coronaphobia and chronophobia, which may be a consequence of everyday life in fear, fear and existential insecurity [20]. At this point, it is necessary to recall the results of studies that confirmed the suicides of medical workers, for instance in India. Virus infection was found to be the most common cause of suicide, followed by work-related stress and stress from COVID-19 infection/transmission. In total, 26 medics aged 22–60 took their own lives [21]. Other studies highlight that people are wary of people experiencing COVID-19, even when they are the closest family members [22]. In view of the above, supporting physicians by society is a very important task on which, to a large extent, the health condition of patients depends. Studies conducted in various places around the world, including Nepal, show that doctors experience symptoms of depression, stress and anxiety of varying severity [23]. Therefore, generating appropriate conditions for reducing emotional tensions in the work of doctors is an indispensable task, the implementation of which will affect their general well-being, including pro-vaccination behavior. This research shows that the attitude of doctors can encourage patients to take the vaccine. American research confirms the thesis that doctors are highly trusted and perceived positively in terms of generating pro-vaccination attitudes [24]. If we can talk about the risks taken by doctors in this matter, it is an important determinant of decisions related to health and the adoption of healthy behaviors and avoiding the opposite [25]. Specialist support is now needed for physicians, especially for those who have had COVID-19 infection. Based on the research carried out on this issue, it can be noted that most health care workers affected by COVID-19 show adaptive emotional and behavioral responses to high levels of stress; therefore, psychotherapy techniques based on the model of adaptation to stress and eliminating unfavorable behavior are useful [26]. On the basis of the presented results, it can be concluded that Polish doctors revealed positive feelings after receiving the vaccine, which may contribute to generating pro-vaccination attitudes, social well-being and building responsible interactions with patients.

The attitude of the Polish health service correlates with the opinion of scientists in the UK who believe that the adoption of the vaccine by doctors is safe and is the most powerful tool to quickly recover from the pandemic and minimize the significant economic, social and psychological impact that the pandemic has given us [27].

## 5. Conclusions

In summary, it should be stated that the results of the conducted research confirm that Polish doctors feel positive after taking the COVID-19 vaccine. It should be assumed that the behavioral attitudes of the respondents may contribute to the generation of similar attitudes among patients. As a result, it may stop the pandemic and reduce social emotional tension, especially anxiety and depression. From a psychosocial point of view, vaccination has a positive effect on the existential safety of doctors and their interactions with patients, although young doctors are not so afraid of infections.

## Figures and Tables

**Table 1:** Mean and standard deviation of age and work experience and number of respondents in particular age groups and for a given gender, and the number of respondents in each group.

KERRYPNX		Age (Years)	Work Experience (Years)
Group of Respondents	*n*	Mean ± SD	Mean ± SD
All respondents	149	32.1 ± 6.99	6.2 ± 6.8
Gender:			
women	85	31.18 ± 6.35	5.25 ± 6
men	64	33.38 ± 7.61	7.48 ± 7.62
Age:			
26–30 years old	93	28.03 ± 1.34	2.55 ± 1.26
31–45 years old	48	36.67 ± 4.18	9.94 ± 4.69
46–69 years old	8	52.38 ± 7.63	26.38 ± 8.63

**Table 2:** Comparison of the answer of the given question with age.

Question	Answer	Age			Chi-Square Test for Independence		
		26–30 yo	31–45 yo	46–69 yo	Chi-Square	df	*p*
		*n*	*n*	*n*			
How do you rate the effectiveness of the vaccine you have received?	Effective	44 (47.3%)	37 (77.1%)	6 (75%)	12.886	4	0.012
	Ineffective	2 (2.2%)	1 (2.1%)	0			
	I have no opinion	47 (50.5%)	10 (20.8%)	2 (25%)			
Are you worried about the negative effects of taking the vaccine?	Yes	21 (22.6%)	6 (12.5%)	1 (12.5%)	14.205	6	0.027
	No	55 (59.1%)	25 (52.1%)	5 (62.5%)			
	I do not think about that	12 (12.9%)	17 (35.4%)	1 (12.5%)			
	I do not care	5 (5.4%)	0	1 (12.5%)			
Do you agree that taking the vaccine will help to contain the pandemic?	I definitely agree	42 (45.1%)	32 (66.7%)	5 (62.5%)	9.837	8	0.277
	I agree	38 (40.9%)	11 (22.9%)	3 (37.5%)			
	I rather agree	9 (9.7%)	3 (6.2%)	0			
	I rather disagree	0	1 (2.1%)	0			
	Hard to say	4 (4.3%)	1 (2.1%)	0			
Did you feel fear when you were vaccinated?	Yes	8 (8.6%)	5 (10.4%)	1 (12.5%)	2.25	4	0.69
	Little	27 (29%)	19 (39.6%)	3 (37.5%)			
	No	58 (62.4%)	24 (50%)	4 (50%)			

**Table 3:** Comparison of the answer of the given question with gender.

Question	Answer	Gender		Chi-Square Test for Independence		
		Women	Men	Chi-Square	df	*p*
		*n*	*n*			
How do you rate the effectiveness of the vaccine you have received?	Effective	43 (50.6%)	44 (68.7%)	4.958	2	0.084
	Ineffective	2 (2.4%)	1 (1.6%)			
	I have no opinion	40 (47%)	19 (29.7%)			
Are you worried about the negative effects of taking the vaccine?	Yes	21 (24.7%)	7 (10.9%)	5.647	3	0.13
	No	45 (52.9%)	40 (62.5%)			
	I do not think about that	17 (20%)	13 (20.3%)			
	I do not care	2 (2.4%)	4 (6.3%)			
Do you agree that taking the vaccine will help to contain the pandemic?	I definitely agree	43 (50.6%)	36 (56,3%)	3.023	4	0.554
	I agree	32 (37.6%)	20 (31.2%)			
	I rather agree	8 (9.4%)	4 (6.2%)			
	I rather disagree	0	1 (1.6%)			
	Hard to say	2 (2.4%)	3 (4.7%)			
Did you feel fear when you were vaccinated?	Yes	6 (7.1%)	8 (12.5%)	3.716	2	0.156
	Little	33 (38.8%)	16 (25%)			
	No	46 (54.1%)	40 (62.5%)			

**Table 4:** Comparison of the number of side effects after taking the vaccine with age and gender.

			Side Effect after Taking the Vaccine								
Variable			Pain at the Injection Site	Fatigue	Headache	Muscle Aches	Arthralgia	Chills	Fever	Nausea	Redness or Swelling at the Injection Site
Age	26–30 yo	yes	88	46	37	36	16	23	20	9	19
	*n*	no	5	47	56	57	77	70	73	84	74
	31–45 yo	yes	44	22	25	22	13	16	12	4	7
	*n*	no	4	26	23	26	35	32	36	44	41
	46–69 yo	yes	8	3	4	5	2	3	6	0	2
	*n*	no	0	5	4	3	6	5	2	8	6
	chi-square test	chi-sq	1.031	0.516	2.052	2.074	1.966	1.519	11.104	0.88	0.923
		df	2	2	2	2	2	2	2	2	2
		*p*	0.597	0.772	0.358	0.354	0.374	0.468	0.004	0.644	0.63
Gender	Women *n*	yes	83	37	40	37	16	27	22	7	18
		no	2	48	45	48	69	58	63	78	67
	men *n*	yes	57	34	26	26	15	15	16	6	10
		no	7	30	38	38	49	49	48	58	54
	chi-square test	chi-sq	4.741	1.348	0.612	0.126	0.471	1.251	0.015	0.060	0.737
		df	1	1	1	1	1	1	1	1	1
		*p*	0.029	0.246	0.434	0.722	0.492	0.263	0.903	0.807	0.391

**Table 5:** Comparison of mean of the afraid of infection and getting sick with the gender.

			Mann–Whitney Test	
Variable	Mean ± SD	*n*	*z*-Value	*p*
Are you afraid of infection and getting sick?				
women	2.69 ± 1.11	85	−3.8621	<0.001
men	3.58 ± 1.41	64		
Are you afraid of infection and sickness of your loved ones?				
women	4.22 ± 0.93	85	−0.8712	0.384
men	4.36 ± 0.84	64		

**Table 6:** Comparison of mean of the afraid of infection and getting sick with age.

			Kruskal–Wallis Test			Mann–Whitney Test					
						AvsB		AvsC		BvsC	
**Variable**	**Mean ± SD**	***n***	**chi-sq**	**df**	***p***	**z-val**	***p***	**z-val**	***p***	**z-val**	***p***
Are you afraid of infection and getting sick?											
26–30 years old	2.62 ± 1.16	93	30.066	2	<0.001	−5.20	<0.001	−2.62	0.009	−0.02	0.982
31–45 years old	3.83 ± 1.21	48									
46–69 years old	3.75 ± 1.49	8									
Are you afraid of infection and sickness of your loved ones?											
26–30 years old	4.15 ± 0.85	93	11.232	2	0.0036	−3.22	0.0013	−0.94	0.349	0.37	0.712
31–45 years old	4.58 ± 0.79	48									
46–69 years old	4.00 ± 1.51	8									

Note: A is “26–30 years old”, B is “31–45 years old”, C is “46–69 years old”.

## Data Availability

Data available on request from the authors.
